# Designing Inclusive HPV Cancer Vaccines and Increasing Uptake among Native Americans—A Cultural Perspective Review

**DOI:** 10.3390/curroncol28050316

**Published:** 2021-09-24

**Authors:** Skyler J. Bordeaux, Anthony W. Baca, Rene L. Begay, Francine C. Gachupin, J. Gregory Caporaso, Melissa M. Herbst-Kralovetz, Naomi R. Lee

**Affiliations:** 1 Department of Health Sciences, Northern Arizona University, Flagstaff, AZ 86011, USA; sjb625@nau.edu; 2 Department of Chemistry and Biochemistry, Northern Arizona University, Flagstaff, AZ 86011, USA; awb243@nau.edu; 3 Centers for American Indian and Alaska Native Health, University of Colorado Anschutz Medical Campus, Aurora, CO 80045, USA; rene.begay@cuanschutz.edu; 4 Department of Family and Community Medicine, College of Medicine, University of Arizona, Tucson, AZ 85711, USA; fcgachupin@arizona.edu; 5 Center for Applied Microbiome Science, Pathogen and Microbiome Institute, Northern Arizona University, Flagstaff, AZ 86011, USA; greg.caporaso@nau.edu; 6 Department of Basic Medical Sciences, The University of Arizona College of Medicine-Phoenix, Phoenix, AZ 85004, USA; mherbst1@arizona.edu; 7 Department of Obstetrics and Gynecology, The University of Arizona College of Medicine-Phoenix, Phoenix, AZ 85004, USA; 8 University of Arizona Cancer Center, Phoenix, AZ 85004, USA

**Keywords:** Native American, HPV, cervical cancer, vaccines, bioethics, clinical trials

## Abstract

Despite a global and nationwide decrease, Native Americans continue to experience high rates of cancer morbidity and mortality. Vaccination is one approach to decrease cancer incidence such as the case of cervical cancer. However, the availability of vaccines does not guarantee uptake, as evident in the Coronavirus 2019 pandemic. Therefore, as we consider current and future cancer vaccines, there are certain considerations to be mindful of to increase uptake among Native Americans such as the incidence of disease, social determinants of health, vaccine hesitancy, and historical exclusion in clinical trials. This paper primarily focuses on human papillomavirus (HPV) and potential vaccines for Native Americans. However, we also aim to inform researchers on factors that influence Native American choices surrounding vaccination and interventions including cancer therapies. We begin by providing an overview of the historical distrust and trauma Native Americans experience, both past and present. In addition, we offer guidance and considerations when engaging with sovereign Tribal Nations in vaccine development and clinical trials in order to increase trust and encourage vaccine uptake.

## 1. Introduction

The term American Indian Alaska Native (AIAN) aligns with governmental agency terminology; however, we more often prefer and engage the terms Native American, Native, and Indigenous. In certain instances, the specific tribal nation name can be used to recognize their tribal status (i.e., Hopi or Cherokee Nation) rather than broad pan-ethnic identities such as Native American [1]. However, other communities also choose to remain anonymous out of distrust or fear of stigmatization. Thus, this review utilizes a critical Indigenous lens [2], as four of the authors self-identity from four unique Native American communities, on a unique and insightful interpretation of cancer vaccine design, specifically human papillomavirus (HPV), related to Native American communities. Our article will discuss four areas: (1) recent reports on cervical cancer and human papillomavirus (HPV) prevalence among Native Americans; (2) historical trauma and medical distrust; (3) considerations to broaden immunity in HPV vaccine design; and (4) best practices for engaging Native American communities in future cancer vaccine trials and uptake.

### 1.1. Cervical Cancer Disparities among Native American Women

Cervical cancer is the leading gynecological malignancy worldwide [3]. HPV infections were implicated in nearly all cervical cancers. However, HPV is associated with other cancers to include vulvar/vaginal, anal, penile, and oropharyngeal. Approximately 13,800 new cases of cervical cancer were estimated in the United States (U.S.) in 2020 [4]. While the cervical cancer mortality rate has declined, approximately 4290 women may still die from this disease annually.

The decline in cervical cancer mortality is due to the utilization of the Papanicolaou (Pap) smear [5]. More recently, the Pap screenings were augmented with the HPV vaccine and hrHPV type-specific testing. However, despite the decline, U.S. cancer registries with linkage to Indian Health Service (IHS) records showed a 25% higher rate of cervical cancer incidence and mortality in Native women when compared with Hispanic non-white women, with a nearly two-fold variation across IHS regions [6,7,8]. Several studies also showed that, for most U.S. regions, cervical cancer mortality rates among Native women are consistently higher than those for all other races [8,9]. The reasons for these disparities are not known but may be due to a higher prevalence of high-risk HPV genotypes and a lower prevalence of screening among the population [10,11].

### 1.2. HPV Prevalence in Native American Communities

**Figure 1:**
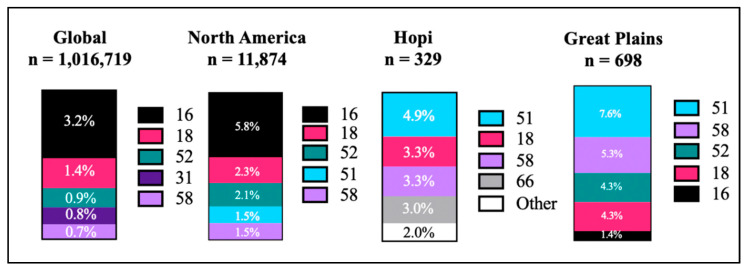
Most prevalent hrHPV types reported in women tested globally [7,12,13].

One analysis included 194 studies comprising of over one million women worldwide, that estimated a global HPV prevalence of 11.7% [12]. However, not all HPV types are the same. Of the over 200+ types identified, there are 14 HPV genotypes known to be oncogenic or high-risk HPV (hrHPV) which includes HPV-16, 18, 31, 33, 35, 39, 45, 51, 52, 56, 58, 59, 66, and 68. The most common hrHPV genotypes globally found are HPV-16, 18, 31, 52, and 58 (Figure 1) [12]. Within North America, the most common types were HPV-16 (5.8%), HPV-18 (2.3%), HPV-52 (2.1%), and HPV-58 & 51 (combined, 1.5%) [12]. Notably, HPV-16 and -18 genotypes were responsible for approximately 70% of all cervical cancers worldwide [12]. In a meta-analysis study of women, the most prevalent hrHPV types were reported globally and by continent [12]. The trends were similar both globally and in North America with HPV-16 as the most prevalent. However, among two separate studies in Native American women, HPV-16 was the least prevalent. Alarmingly, HPV-51 was the most prevalent among Native women and is not covered by the current HPV vaccines [7,13]. Since the introduction of the HPV vaccines, hrHPV prevalence decreased among women eligible for vaccination, specifically for HPV-16 and -18 [14]. Native American women were insufficiently sampled to permit a population-specific estimate of hrHPV prevalence or impact of vaccination [7,15,16].

Based on recent studies, hrHPV genotypes are affecting Native American women more compared to non-Native women. Two separate studies reported higher prevalence of hrHPV compared to the national average among Native women ages 21–65 years. In Hopi women (*n* = 329) from Arizona, it was estimated that approximately 22% were positive for at least one hrHPV [13]. More specifically, the four most prevalent HPV-51, -18, -58, -66 types were found among Hopi women (Figure 1). Meanwhile, 35% of the women (*n* = 698) from a single tribe in the Great Plains were positive for at least one hrHPV [7]. Among this population the most prevalent were HPV-16, -18, -51, -52, and -58. Most notably, HPV-51 was found in 4.9% of Hopi women and 7.6% of Native women sampled from the Great Plains [7,13]. While HPV-51 is not one of the leading types detected in cervical cancers from the general population, the most prevalent type across Native populations is unknown.

The prevalence of hrHPV types affected by vaccines continues to decline in women ages 14–24 years [17]. Comparatively, women aged 21–24 years that were positive for at least one hrHPV type was 38% and 42.2% in the Hopi and Great Plains women, respectively [7,13]. In the general population, the hrHPV prevalence declined across age groups [18]. Unfortunately, the decline was not as significant in Native women. Among Hopi women, there was a decline from 38% in women 21–24 years old to 14% in women over 50 years of age [13]. However, a smaller decline from 42.2% in women 21–24 years old to 27.9% in women 50–65 years old was seen in Native women from the Great Plains [7]. Furthermore, HPV-16 and -18 had the lowest prevalence among Native women ranging from 21–24 years old in both studies. Prior to the HPV vaccines, these two types were typically the most common. Thus, the lack of prevalence in young women is the only evidence that vaccines are successfully protecting against HPV among Native women. These findings indicate that overall prevalence of the 14 hrHPV types assessed are higher in Native American women than in the general U.S. population, especially among women aged 50–65 years. Unfortunately, the HPV prevalence was not assessed in any of the 574 federally recognized tribal communities since the introduction of Gardasil 9^®^ [7,13].

## 2. Historical Trauma and Medical Distrust among Native American Populations

### 2.1. History of Medical Distrust

**Figure 2:**
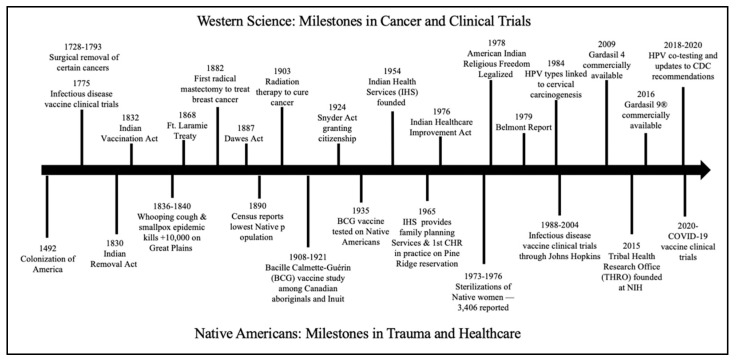
Cancer and clinical trial milestones in western history in relation to Native American experiences.

In Figure 2, we highlight government actions and milestones relevant to Native American communities and health, including those for cancer and clinical trials. We also provide context for today’s distrust among Native Americans.

Native American communities are resistant to participate in research studies for many reasons: (1) not wanting to be “guinea pigs”; (2) findings are not uniformly shared or transparency is lacking; (3) findings do not necessarily increase local capacity, including prevention, sustained intervention, and/or services; (4) benefits are not maximized; and (5) ability to participate (e.g., transportation or child care) are not optimized [19]. These concerns stem from historical experiences, for example, healthcare providers have undermined the well-being of Native women by pathologizing holistic views of health and by general patronizing [17]. Further, Native women have been restricted access to healthcare and the right to bear and raise children [20], as evident in the sterilization of Native women without informed consent in the 1970s [19]. Broader government policies were designed to annihilate Native Americans in the 19th and early 20th centuries

In 2018, Native women diagnosed with cancer were surveyed and interviewed about their healthcare experiences. Results showed that many women struggled to be heard, to be taken seriously, or to have their legitimate healthcare concerns addressed; over 41% (*n* = 18) reported struggling to have their initial concerns about cancer taken seriously and women consistently reported having to convince their doctors that something was wrong [20]. Women who were identified as having cancer early on usually reported finding out they had cancer through regular screening programs, highlighting the importance of these programs [20]. In the U.S., cervical cancer incidence is nearly as twice as high in counties with poverty levels >20% compared to those with poverty levels >10%, cervical cancer incidence and mortality rates are 25% and 95% higher, respectively. According to the 2018 U.S. Census, Native Americans had the highest poverty rate at 25.4% while the white population had a poverty rate of 10.1% [21]. This correlation implies a greater incidence of cervical cancer will be seen among Native Americans strengthening the importance of regular screening programs.

Challenges reported by patients (22%, *n* = 10) included a lack of consistency in care providers, an insufficient number of providers, and inexperienced healthcare professionals, all of which participants identified as being barriers for their cancer treatment [20]. Several patients reported problems with not receiving results or information because there was not an available doctor [20]. Positive experiences were important for women to be able to continue their treatment. These experiences included nurses and doctors spending time to communicate with their patients and providing emotional support; negative experiences included disrespectful comments and insufficient communication. Many Native women experienced negative and insensitive care and treatment from their healthcare providers; this was a source of considerable distress.

### 2.2. Sterilization of Native American Women

Although female sterilization can be an empowering method of contraception, it has negative history as a method used to eugenically control and marginalize minority populations, including Native American women [22]. The U.S. government agency personnel targeted Native Americans for family planning because of their high birth rate [23]. In 1955, Congress transferred total responsibility for Native health from the Department of Interior (formerly the Department of War) to the Public Health Service (PHS). The legislation stated that, “All facilities transferred shall be available to meet the health needs of the Indians and that such health need shall be given priority over that of the non-Indian population.” The PHS, a division of the Department of Health, Education, and Welfare (HEW), formed the Division of Indian Health, which was renamed the Indian Health Service (IHS) in 1958 [23]. The IHS began providing family planning services for Native Americans in 1965 under the authority of HEW and PHS.

The 1970 census revealed that the average Native woman bore 3.79 children, whereas the median for all groups in the U.S. was 1.79 children [23]. Protests and investigations that emerged in 1970s showed that the public health system, primarily the IHS, was sterilizing Native American women without their knowledge or informed consent [24]. In 1973, the HEW proposed deterrent regulations on sterilization procedures. One of the regulations stated that competent individuals must grant their informed consent, that there must be a signed consent form in the possession of the agency performing the sterilization showing that the patient understands the benefits and the costs of sterilizations, and that a 72-h waiting period must occur between the time of consent and the surgical procedure. Another regulation established a moratorium on sterilization persons under the age of 21 and those whom doctors had declared mentally incompetent [23]. However, the General Accounting Office (GAO) investigation found that the weakness in HEW regulations involving the inadequate documentation of consent forms could be attributed to the failure of IHS area offices to adhere to new HEW sterilization regulations and failure to adopt proper consent forms. In fact, of 113 voluntary sterilizations reviewed in Aberdeen, Phoenix, and Oklahoma City, none were in full compliance with new HEW regulations [24]. Contract clinical facilities also failed to meet HEW requirements for consent forms, allowing room for coercion and misinformed consent [24]. Without evidence documenting informed consent, it is possible that procedures were falsely presented as necessary sterilizations.

**Figure 3:**
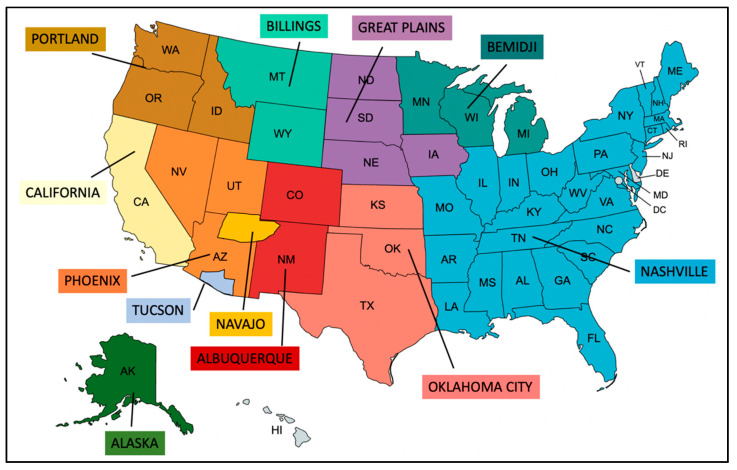
Twelve Indian Health Service areas.

Hospital staff and sterilized women began to speak out about the sterilization abuse and other problems within the IHS and PHS. Investigations into sterilization abuse arose from an inquiry made by Dr. Connie Uri and Senator James Abourezek of South Dakota [24]. In 1976, the GAO investigated 4 of 12 areas serviced by the IHS (Figure 3): Aberdeen, South Dakota (now Great Plains); Albuquerque, New Mexico; Oklahoma City, Oklahoma; and Phoenix, Arizona. The GAO investigators examined 3406 sterilizations performed by IHS during the fiscal years of 1973–1976. These numbers did not include those conducted in the Albuquerque area because contract physicians performed all sterilizations in that IHS region and were unable to determine the exact number. IHS was also unable to provide information concerning which procedures were performed for therapeutic purposes or for non-therapeutic birth control purposes [24]. In many cases of uninformed sterilizations, consent forms were signed while the patient was anesthetized or in the throes of labor; many of these women did not recall signing the form yet their signatures were present [24]. After examining just a three-year period (1973–1976), the GAO identified 3406 involuntary sterilizations performed on Native women aged 15–44 years [25]. GAO personnel did not interview any Native American women who had been sterilized during this period because they said they, “believe[d] that such an effort would not have been productive [23].” Failure to interview the sterilized women meant investigators could not fully comprehend the possibilities of coercion encountered by these women or the impact of weaknesses in regulations [24].

Prompted by the numerous charges, investigations, and lawsuits in the early 1970s, the HEW announced another round of regulations regarding sterilizations that attempted to address and prevent coercion, effective on 8 March 1979. The procedures for sterilizations were changed in the following ways: (1) the waiting period is now 30 days rather than 72 h; (2) new consent forms use simpler language; (3) an language interpreter must be present; (4) the distinction must be made between medical (therapeutic) and family planning (non-therapeutic) sterilizations; and (5) no federal money can be used or provided for a hysterectomy without medical reason or on an individual under 21 years [24]. These IHS practices harmed the relationships between the U.S. government and tribal families and communities. The operations also caused an inordinate amount of harm to the individual Native American women sterilized by IHS physicians [23]. The lasting legacy of sterilization abuse is a missing generation of Native American children who may have learned and passed down tribal traditions, ceremonies, and language, and continued the fight for cultural and political self-determination [24].

### 2.3. Bioethical Misconduct toward Native Americans

In 2004, the Havasupai Tribe filed a lawsuit against the Arizona Board of Regents (ABOR) and Arizona State University (ASU) researchers upon discovering their DNA sample, initially collected for genetic studies on type 2 diabetes, were misused [26]. Between 1990 and 1994, DNA samples were solicited from approximately 400 Havasupai tribe members in conjunction with the Diabetes Project led by researchers at ASU. The stated intent of the project was to understand why more than half of the Havasupai adults suffered from type 2 diabetes [26]. The researchers obtained Institutional Review Board (IRB) approval from ASU for studies on diabetes and schizophrenia; however, Havasupai participants alleged that researchers had failed to make clear that samples may be used for studies on schizophrenia and that no expanded informed consent was sought. This was especially concerning since mental illness was highly stigmatized and tribal members asserted they would have not consented to such research [26]. The biosamples were used to study schizophrenia but also inbreeding and population migration, which have not benefitted the tribe in any way [25]. In April 2010, the *Havasupai v. Arizona Board of Regents* case reached a settlement and the tribe received financial compensation and the DNA samples were returned. The return of DNA samples was a significant moment for the Havasupai Tribe because DNA and biological materials are considered sacred to the tribe which is how many other Native American communities view their biospecimens [26]. It is important to note that there was no court ruling and the case was dismissed due to a procedural error, resulting in no legal precedent and leaving ambiguity over how the informed consent forms should have been interpreted [27].

## 3. Vaccine Design and Formulation

### 3.1. Vaccine Uptake Is High among Native American Youth

In 2019, the Center for Disease Control and Prevention (CDC) modified HPV recommendations to two doses of Gardasil 9^®^ for all individuals aged 9–26 years; previously males were only recommended until 21 years old. IHS data indicate that Native American youth (13–17 years) are in greater compliance with HPV vaccination than the general population. For example, one dose of the HPV vaccine was greater than 80% in both Native American males and females in comparison to 73.2% and 69.8% for general U.S. females and males, respectively [28].

Unfortunately, Native Americans were not included in vaccine impact studies since the introduction of Gardasil 9^®^. Therefore, any indication the vaccine is reducing hrHPV prevalence and cervical cancer rates is unknown [7]. As we design new HPV and cancer vaccines, the medical and research community must include accurate representation of Native Americans and other underrepresented racial/ethnic groups during vaccine trials in order to increase coverage and evaluate efficacy [19]. Otherwise, vaccines designed for the general population may not be as efficacious in populations excluded from the clinical trials, as suggested by the prevalence of hrHPV types among two Native American tribes not included in the current U.S. Food and Drug Administration (FDA) approved vaccine (Figure 1). Additional studies of the prevalence of hrHPV types among other Native American tribes are warranted and may inform future vaccine development.

### 3.2. The General Population Does Not Always Represent the Whole

The current available HPV vaccine (Gardasil 9^®^) protects against seven hrHPV types, including HPV-16, 18, 31, 33, 45, 52, and 58. Of which, four were the most prevalent in North America (Figure 1). In addition, Gardasil 9^®^ protects against two low-risk HPV (lrHPV) types associated with genital warts (HPV-6 and -11). It is estimated that individuals vaccinated with Gardasil 9^®^ will be protected against 90% of hrHPV-associated cancers and 90% of genital warts [16]. However, these estimates are based on the HPV types most prevalent in the general population that did not over sample for Native Americans.

The most prevalent hrHPV types in cervical cancers among Native American women has not been well studied; however, two studies suggest that other hrHPV genotypes not represented in the current FDA-approved vaccines are found frequently in these populations [7,13]. Therefore, future HPV vaccines must provide broader coverage for types not found in the general population. For example, coverage against HPV-51 is required as it is the most prevalent in two geographically separated Native American tribes (Figure 1) [7,13]. In addition, the need for a therapeutic vaccine is evident due to the high hrHPV prevalence among older Native women who are most at-risk [7,13]. Finally, as future vaccine sponsors consider clinical trials or new FDA approvals, it is imperative that the vaccine formulation is transparent. Misconceptions and misunderstanding surrounding vaccine formulation may cause hesitancy in vaccine uptake [9]. For example, cultural concerns were raised by traditional healers and medicine men regarding the recent Coronavirus 2019 (COVID-19) vaccines [29]. It is important that vaccine sponsors and government agencies are aware of such concerns and provide more transparency and work equitably with sovereign Tribal Nations, in order to stop the spread of false information and increase vaccine acceptance.

## 4. Recommendations for Increasing Native American Representation in Vaccine Trials and Uptake

The COVID-19 pandemic underscored the hesitancy and risks experienced by Native American populations. Research that appropriately addresses distrust and maximizes benefits among the Native people will they espouse the scientific and clinical approaches available [30,31,32]. Current SARS-CoV-2 vaccine trial enrollments emphasize the underrepresentation of diverse races and ethnicities. For example, in the Moderna Phase III COVID-19 vaccine trials, 79% of participants were white and 0.8% were Native American [33]. However, recruitment of Native Americans was slow until the implementation of the COVID-19 Prevention Network (CoVPN) and advisement from the Native and Indigenous Expert Panelists [34].

**Figure 4:**
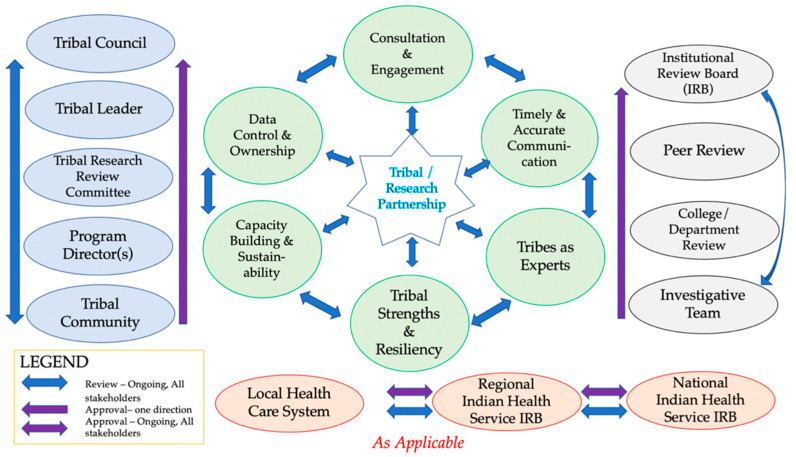
Recommendations for best practices to increase Native American representation in vaccine trials or other research.

To increase successful engagement of sovereign Tribal Nations in cancer vaccine clinical trials and research, several best practices and approaches are strongly recommended to optimize benefit, ensure strong partnerships, and contribute to generalizable knowledge [35]. These best practices are not mutually exclusive or require a one-time effort; they are interrelated and ongoing over the duration of the partnership. The best practices include: (1) consultation and engagement; (2) tribal data control and ownership; (3) timely and accurate communications; (4) capacity building and sustainability; (5) acknowledgement of tribes as experts; and (6) contribution to tribal strengths and resiliency (see Figure 4) [36].

### 4.1. Consultation and Engagement

The focus, question, goal, hypothesis, and objectives of a research study should be jointly determined for true tribal consultation and engagement to apply. The COVID-19 pandemic prioritized public health and health promotion among Tribal Nations and the survival of their respective communities is of paramount importance. For vaccine trials, the investigator or pharmaceutical company should initiate tribal partnerships long before presentation of the research protocol. A clear and understandable description of the clinical trial should be presented including therapeutic misconception [37]. For example, the vaccine trial is being conducted to determine if the medicine or procedure under study may or may not help in the care of future cancer patients. The main purpose is not to help the present community by participating in trials, although it might. Sometimes people think that the research is being conducted to help themselves; this is called therapeutic misconception.

#### Tribal Review, Approval & Dissemination

Most tribes established procedures and processes for reviewing proposed research and these need to be followed. These reviews and approvals are in addition to any university or commercial IRB. As sovereign nations, tribes rarely defer to other IRBs. Furthermore, if any IHS personnel, facilities, data, or services are involved, approval and review from the Regional and National IHS IRB are also required [38]. For a vaccine trial, the local tribal or urban health service, or IHS will need to be included.

The tribal review and approval of the vaccine trial is critical as their assessment of harms and benefits will be unique to their community and tribal members. Evidence of this approval is often a binding document known as a tribal resolution, which specifies the project details. Oftentimes, a tribe will also require a memorandum of understanding or agreement (MOU/MOA) and data sharing agreement. The MOU/MOA includes details of space, recruitment, sampling, compensation, duration, roles and responsibilities, key stakeholders, data specifications, publication agreements (including presentations), and termination terms [39,40]. If a researcher is working with more than one tribe, review and approval must be sought and obtained from each tribe. No public dissemination of results should occur with the general or scientific community without prior review and approval by the tribe.

### 4.2. Tribal Data Control and Ownership

In true recognition of tribal sovereignty, data garnered as part of the research partnership belongs to the tribe. Furthermore, any sharing or use of data beyond original specific aims must be cleared and allowed by the tribe. This would include any additional data sharing or secondary data use. There should be candid discussions for location of data, duration of data storage, access, disposition, and any disposal understandings. It must be understood that the tribe owns their data and should be treated as such regardless of any outside entities’ rules, regulations, or procedures. For biospecimens, it is within the rights of a tribe to request cultural ceremonies as part of the disposal process, and it is incumbent upon the researcher to ensure biohazards are handled appropriately. If the tribe terminates the project, it is important to discuss the retention or destruction of data and to agree on terms and conditions [41]. It is not ethical to continue to hold on to data, conduct data analysis, or publish research results when a tribe relinquished approval. For clinical trials which often involve sophisticated data elements over extended periods of time, it is recommended that researchers consider the engagement of Native-owned and operated biorepositories as an option [42].

### 4.3. Timely and Accurate Communications

The communications with the tribe should always be in lay terms and avoid jargon or acronyms. Researchers should volunteer visual aids (e.g., illustrations of methods and procedures) for everything being proposed. It is strongly recommended to include a lab and clinic tour to demonstrate steps and introduce personnel. The tribe should be updated on progress on a regular basis, without the researcher having to be reminded. Tribes are oftentimes limited in personnel, space, and time. Researchers should be prepared and have the necessary materials organized. The burden of proof should be shouldered by the researcher, including engagement and inclusion of facilitators or experts in each area. For a vaccine trial, which are often multi-center based, it is important to provide tribes with ongoing data safety, adverse or reportable events, audit reports and findings, and status of the overall trial.

### 4.4. Capacity Building and Sustainability

An essential component of tribal sovereignty is self-governance. Vaccine trials have the potential to build tribal research capacity by which Indigenous scientists can lead research that is directly aligned with community needs and values which are essential for long-term culturally sustainable science [43]. Deliberate actions should be undertaken to enhance the skills, knowledge and understanding of the research for tribes, especially among their students by involving them as research team members. It is recommended that researchers develop tool kits or models that can be used as resources or referenced, after the research concluded. Researchers should also share their networks that may benefit the tribal community and expand the capacity and capability of tribal entities.

Tribes are oftentimes limited in personnel, space, time, and capacity to sustain or maintain research specific aims; therefore, researchers should include activities where the tribe can benefit directly from their involvement with the research project as well as the researcher. For example, if a tribe were asked to participate in SARS-CoV-2 vaccine trial, and the tribe does not have local experts in protocol specifics, the researcher can secure a consultant to help the tribe with its determination. A vaccine science expert, unaffiliated with the research, can be asked to help the tribe to assess the adequacy of the research plan, including the earliest animal testing and into phase I, II, and III trials. Information is therefore needed for a tribe to determine next steps. Another example is to bring in Indigenous science experts from the community being studied or sought by nearby such as the CoVPN Native an Indigenous Expert panel [34].

### 4.5. Acknowledgement of Tribes as Experts

This involves building a framework to engage Indigenous peoples and communities in clinical trials, much like the one developed for genomic research [43]. The goal of this framework is to build trust, increase inclusion of diverse groups in research, and enhance ethical research practices that promote tribal research regulations (e.g., tribal oversight and consultation) and benefits to participants and their communities. The framework should extend beyond the current U.S. federal requirements for biomedical and behavioral research. Successfully completing the study is more likely when relationships are built with tribes and community members and recognizing their expertise increases the possibility of developing subsequent long-term partnerships. The collaborations will build trust and can enhance research participation throughout the study’s duration.

### 4.6. Contribution to Tribal Strengths and Resiliency

Native Americans are the first human inhabitants of North America. They were here for over 10,000 years and represent unparalleled views, strength, and resilience. It is important for researchers to understand these experiences, although varied, weigh into present day priorities. For many tribes, it is not merely health disparity, inequity, inclusion, or human rights. It is about survival as well as the safety and wellbeing of their people and their ability to exist as a people. The tribal experience is expansive, and the tribe is the most informed on what works best for them. In any kind of research, especially one that deals with sensitive topics or biospecimens, it is very important to engage respectfully. The more the research foci address these research priorities and recognize the past historical and current harms, the more a tribe may consider participation.

### 4.7. Importance of Vaccination and Cancer Prevention Engagement

As in any positive human experience, building strong relationships is essential when communicating with tribes about the importance of cancer prevention. Rapport and trust are essential, especially when the conversation focuses on the safety and efficacy of HPV or other vaccines in preventing cancer, to ensure that the benefits of vaccination outweigh any potential risk of side effects. It is only through consistent and comprehensive messaging which tribal leaders fully understand that community-based endorsement and implementation will occur. Tribal leaders will appreciate it if baseline data are presented and clear, and if objective, specific, and time-sensitive approaches are presented with follow-up reporting on a regular basis. It is through timely and accurate data reporting that tribes will be fully engaged and supportive of vaccination, especially among youth, both male and female. Researchers are encouraged to share results of research efforts to the community-at-large so that there is community level ownership and responsibility for disease prevention and health promotion. These community open forums would need tribal clearance and approval of presentation materials beforehand.

As discussed, the negative experiences of tribes faced in trials included not being recognized as sovereign Nations, unapproved secondary data use, naming of tribes resulting in decades-long stigmatization, dissemination of results without prior review or approval, engagement of practices that caused direct harm, etc. Research is often seen as a means to advance academic careers, garner funding for institutions, and publish harmful findings with little to no direct benefit to tribal entities. It is only through demonstration of true participatory research that holds optimized direct benefit for tribes that future vaccine trials will be received favorably.

## 5. Conclusions

The COVID-19 pandemic taught society that development of an effective vaccine does not guarantee positive uptake. Despite their exclusion from past and ongoing clinical trials, Native Americans continue to have high vaccination rates across the nation. Therefore, as we consider future cancer vaccines, researchers and pharmaceutical companies should consider the historical distrust and research harms experienced by one of the most at-risk populations for cancer.

## Data Availability

No new data were created or analyzed in this study. Data sharing is not applicable to this article.

## References

[1] Horse P.G., Wijeyesinghe C.L., Jackson B.W. (2001). Reflections on American Indian identity. New Perspectives on Racial Identity Development: A Theoretical and Practical Anthology.

[2] Garrison N.A., Hudson M., Ballantyne L.L., Garba I., Martinez A., Taualii M., Arbour L., Caron N.R., Rainie S.C. (2019). Genomic Research Through an Indigenous Lens: Understanding the Expectations. Annu. Rev. Genom. Hum. Genet..

[3] Bell M.C., Schmidt-Grimminger D., Patrick S., Ryschon T., Linz L., Chauhan S.C. (2007). There is a high prevalence of human papillomavirus infection in American Indian women of the Northern Plains. Gynecol. Oncol..

[4] National Cancer Institute Cancer Stat Fact Sheets: Cervical Cancer. https://seer.cancer.gov/statfacts/html/cervix.html.

[5] Alfonsi G.A., Datta S.D., Mickiewicz T., Koutsky L.A., Ghanem K., Hagensee M., Kerndt P., Hsu K., Weinstock H., Shlay J.C. (2011). Prevalence of high-risk HPV types and abnormal cervical cytology in American Indian/Alaska Native women, 2003–2005. Public Health Rep..

[6] Jeudin P., Liveright E., Del Carmen M.G., Perkins R.B. (2014). Race, ethnicity, and income factors impacting human papillomavirus vaccination rates. Clin. Ther..

[7] Lee N.R., Winer R.L., Cherne S., Noonan C.J., Nelson L., Gonzales A.A., Umans J.G., Buchwald D., Canc C.I.N. (2019). Human Papillomavirus Prevalence Among American Indian Women of the Great Plains. J. Infect. Dis..

[8] Melkonian S.C., Weir H.K., Jim M.A., Preikschat B., Haverkamp D., White M.C. (2021). Incidence of and Trends in the Leading Cancers With Elevated Incidence Among American Indian and Alaska Native Populations, 2012–2016. Am. J. Epidemiol..

[9] Duvall J., Buchwald D. (2012). Human Papillomavirus Vaccine Policies Among American Indian Tribes in Washington State. J. Pediatr. Adolesc. Gynecol..

[10] Leyden W.A., Manos M.M., Geiger A.M., Weinmann S., Mouchawar J., Bischoff K., Yood M.U., Gilbert J., Taplin S.H. (2005). Cervical cancer in women with comprehensive health care access: Attributable factors in the screening process. J. Natl. Cancer Inst..

[11] Bell M.C., Schmidt-Grimminger D., Jacobsen C., Chauhan S.C., Maher D.M., Buchwald D.S. (2011). Risk factors for HPV infection among American Indian and white women in the Northern Plains. Gynecol. Oncol..

[12] Bruni L., Diaz M., Castellsague X., Ferrer E., Bosch F.X., de Sanjose S. (2010). Cervical human papillomavirus prevalence in 5 continents: Meta-analysis of 1 million women with normal cytological findings. J. Infect. Dis..

[13] Winer R.L., Noonan C.J., Gonzales A.A., Cherne S., Buchwald D.S. (2016). Assessing acceptability of self-sampling kits, prevalence, and risk factors for human papillomavirus infection in American Indian women. J. Community Health.

[14] Lei J., Ploner A., Elfstrom K.M., Wang J., Roth A., Fang F., Sundstrom K., Dillner J., Sparen P. (2020). HPV Vaccination and the Risk of Invasive Cervical Cancer. N. Engl. J. Med..

[15] Fajardo-Bernal L., Aponte-Gonzalez J., Vigil P., Angel-Muller E., Rincon C., Gaitan H.G., Low N. (2015). Home-based versus clinic-based specimen collection in the management of Chlamydia trachomatis and Neisseria gonorrhoeae infections. Cochrane Database Syst. Rev..

[16] Hung M., Su S., Hon E.S., Licari F.W., Park J., Bounsanga J., Tuft J., Otrusinik S., Lipsky M.S. (2021). Health Disparities Associated with Females Reporting Human Papillomavirus Infection in the United States. Womens Health Rep..

[17] Oliver S.E., Unger E.R., Lewis R., McDaniel D., Gargano J.W., Steinau M., Markowitz L.E. (2017). Prevalence of Human Papillomavirus Among Females After Vaccine Introduction-National Health and Nutrition Examination Survey, United States, 2003–2014. J. Infect. Dis..

[18] Wheeler C.M., Hunt W.C., Cuzick J., Langsfeld E., Pearse A., Montoya G.D., Robertson M., Shearman C.A., Castle P.E. (2013). A Population-based Study of HPV Genotype Prevalence in the United States: Baseline Measures Prior to Mass HPV Vaccination. Int. J. Cancer.

[19] Petereit D.G., Burhansstipanov L. (2008). Establishing trusting partnerships for successful recruitment of American Indians to clinical trials. Cancer Control.

[20] Liddell J.L., Burnette C.E., Roh S., Lee Y.S. (2018). Healthcare barriers and supports for American Indian women with cancer. Soc. Work Health Care.

[21] Povertyusa The Population of Poverty USE. https://www.povertyusa.org/facts.

[22] Cackler C.J., Shapiro V.B., Lahiff M. (2016). Female Sterilization and Poor Mental Health: Rates and Relatedness among American Indian and Alaska Native Women. Womens Health Issues.

[23] Lawrence J. (2000). The Indian Health Service and the sterilization of Native American women. Am. Indian Q..

[24] Carpio M. (2004). The Lost Generation: American Indian Women and Sterilization Abuse. Soc. Justice.

[25] Pacheco C.M., Daley S.M., Brown T., Filippi M., Greiner K.A., Daley C.M. (2013). Moving forward: Breaking the cycle of mistrust between American Indians and researchers. Am. J. Public Health.

[26] Garrison N.A. (2013). Genomic Justice for Native Americans: Impact of the Havasupai Case on Genetic Research. Sci. Technol. Hum. Values.

[27] Garrison N.A., Cho M.K. (2013). Awareness and Acceptable Practices: IRB and Researcher Reflections on the Havasupai Lawsuit. AJOB Prim. Res..

[28] Walker T.Y., Elam-Evans L.D., Yankey D., Markowitz L.E., Williams C.L., Fredua B., Singleton J.A., Stokley S. (2019). National, Regional, State, and Selected Local Area Vaccination Coverage Among Adolescents Aged 13–17 Years—United States, 2018. MMWR Morb. Mortal. Wkly. Rep..

[29] Sevigny M. For Native People, Coronavirus Vaccine Trial Raises Specter of Past Traumas. https://www.knau.org/post/native-people-coronavirus-vaccine-trial-raises-specter-past-traumas.

[30] Flores L.E., Frontera W.R., Andrasik M.P., Del Rio C., Mondriguez-Gonzalez A., Price S.A., Krantz E.M., Pergam S.A., Silver J.K. (2021). Assessment of the Inclusion of Racial/Ethnic Minority, Female, and Older Individuals in Vaccine Clinical Trials. JAMA Netw. Open.

[31] LaVallie D.L., Wolf F.M., Jacobsen C., Buchwald D. (2008). Barriers to cancer clinical trial participation among native elders. Ethn. Dis..

[32] Sprague D., Russo J., LaVallie D.L., Buchwald D. (2013). Barriers to Cancer Clinical Trial Participation Among American Indian and Alaska Native Tribal College Students. J. Rural Health.

[33] Baden L.R., El Sahly H.M., Essink B., Kotloff K., Frey S., Novak R., Diemert D., Spector S.A., Rouphael N., Creech C.B. (2021). Efficacy and Safety of the mRNA-1273 SARS-CoV-2 Vaccine. N. Engl. J. Med..

[34] COVPN COVPN Community and Stakeholder Engagement Strategic Plan. https://www.coronaviruspreventionnetwork.org/files/covpn-community-stakeholder-engagement-strategic-plan.pdf.

[35] Vigil D., Sinaii N., Karp B. (2021). American Indian and Alaska Native Enrollment in Clinical Studies in the National Institutes of Health’s Intramural Research Program. Ethics Hum. Res..

[36] Gachupin F., Molina F. (2019). How to Conduct Research in American Indian and Alaska Native Communities.

[37] Gachupin F., Tracy K., Slowtalker J. (2020). Clinical Trials, What Are They?.

[38] IHS Human Subjects Research Protections. https://www.ihs.gov/dper/research/hsrp/.

[39] Beans J.A., Saunkeah B., Brian Woodbury R., Ketchum T.S., Spicer P.G., Hiratsuka V.Y. (2019). Community Protections in American Indian and Alaska Native Participatory Research-A Scoping Review. Soc. Sci..

[40] Gachupin F.C., Jenny R., Joe J.R., Gachupin F.C. (2012). Protections to consider when engaging American Indians/Alaska Natives in human subjects research. Health and Social Issues of Native American Women.

[41] Gachupin F.C., Freeman W.L., Solomon T.G.A., Randall L.L. (2014). Chapter 11—Ethics of Biospecimin Research. Conducting Health Research with Native American Communities.

[42] NBDC Research for Natives, By Natives. http://www.nativebio.org/.

[43] Claw K.G., Anderson M.Z., Begay R.L., Tsosie K.S., Fox K., Garrison N.A., Nanibaa’A G., Summer Internship for INdigenous Peoples in Genomics (SING) Consortium (2018). A framework for enhancing ethical genomic research with Indigenous communities. Nat. Commun..

